# Successful Immunomodulatory Treatment of COVID-19 in a Patient With Severe ACTH-Dependent Cushing’s Syndrome: A Case Report and Review of Literature

**DOI:** 10.3389/fendo.2022.889928

**Published:** 2022-06-22

**Authors:** Bojana Popovic, Aleksandra Radovanovic Spurnic, Jelena Velickovic, Aleksandra Plavsic, Milica Jecmenica-Lukic, Tijana Glisic, Dusan Ilic, Danka Jeremic, Jelena Vratonjic, Vladimir Samardzic, Zoran Gluvic, Tatjana Adzic-Vukicevic

**Affiliations:** ^1^ Covid Hospital Batajnica, University Clinical Centre of Serbia, Belgrade, Serbia; ^2^ Clinic for Endocriniology, Diabetes, and Metabolic Diseases, University Clinical Centre of Serbia, Belgrade, Serbia; ^3^ School of Medicine, University of Belgrade, Belgrade, Serbia; ^4^ Clinic for Infectious and Tropical Diseases, University Clinical Centre of Serbia, Belgrade, Serbia; ^5^ Centre for Anesthesiology and Reanimation, University Clinical Centre of Serbia, Belgrade, Serbia; ^6^ Clinic for Allergology and Immunology, University Clinical Centre of Serbia, Belgrade, Serbia; ^7^ Clinic for Neurology, University Clinical Centre of Serbia, Belgrade, Serbia; ^8^ Clinic for Gastroenterology and Hepatology, University Clinical Centre of Serbia, Belgrade, Serbia; ^9^ Clinic for Internal Medicine, Department of Endocrinology and Diabetes, Zemun Clinical Hospital, Belgrade, Serbia; ^10^ Clinic for Pulmology, University Clinical Centre of Serbia, Belgrade, Serbia

**Keywords:** Cushing’s syndrome, COVID-19, IVIg, hypercortisolism, immunomodulation, immunosuppression

## Abstract

**Introduction:**

Patients with Cushing’s syndrome (CS) represent a highly sensitive group during corona virus disease 2019 (COVID-19) pandemic. The effect of multiple comorbidities and immune system supression make the clinical picture complicated and treatment challenging.

**Case report:**

A 70-year-old female was admitted to a covid hospital with a severe form of COVID-19 pneumonia that required oxygen supplementation. Prior to her admission to the hospital she was diagnosed with adrenocorticotropic hormone (ACTH)-dependent CS, and the treatment of hypercortisolism had not been started yet. Since the patient’s condition was quickly deteriorating, and with presumend immmune system supression due to CS, we decided on treatement with intraveonus immunoglobulins (IVIg) that enabled quick onset of immunomodulatory effect. All comorbidities were treated with standard of care. The patient’s condition quickly stabilized with no direct side effects of a given treatment.

**Conclusion:**

Treatment of COVID-19 in patients with CS faces many challenges due to the complexity of comorbidity effects, immunosupression and potential interactions of available medications both for treatment of COVID-19 and CS. So far, there are no guidelines for treatment of COVID-19 in patients with active CS. It is our opinion that immunomodulating therapies like IVIg might be an effective and safe treatment modality in this particularly fragile group of patients.

## Introduction

Dealing with corona virus disease 2019 (COVID-19) focused medical attention on several sensitive population groups. While the knowledge is still improving, some of the recognized risk factors for severe form of the disease are male sex, older age, obesity, hypertension, diabetes mellitus, and cardio-vascular disease (1). This constellation of morbidities is particularly intriguing from endocrine point of view, since they are all features of patients with Cushing’s syndrome (CS). Another relevant feature of CS is a propensity for infections due to profound immune suppression, with prevalence of 21-51%; even more so, infections are the second cause of death (31%) in CS after disease progression, and are the main cause of death (37%) in patients who died within 90 days of diagnosis (2).

Immune system alterations in CS lead to depression of both innate and adaptive immune responses, favoring not only commonly acquired but also opportunistic bacterial infections, fungal infections, and severe, disseminated viral infections (3). Susceptibility to infections directly positively correlates with cortisol level, and is more frequent in ectopic ACTH secretion (EAS). Hypercortisolism hampers the first-line response to external agents and consequent activation of the adaptive response (3). Consequently, there is a decrease in total number of T-cells and B-cells, as well as a reduction in T-helper cell activation, which might favor opportunistic and intracellular infections. On the other hand, an increase in pro-inflammatory cytokine secretion, including interleukine-6 (IL-6) and tumor necrosis factor-α (TNF-α) leads to persistent, low-grade inflammation. It is important to note that immune system changes are confirmed both during the active phase and while in remission of CS (3).

In view of the aforementioned data, a few topics emerge regarding patients with CS and COVID-19. Initial clinical presentation may be altered – low-grade chronic inflammation and poor immune reaction might limit febrile response in the early phase of infection, aggravating timely diagnosis (4). Increased cytokine levels may put patients with CS at increased risk of severe course and progression to acute respiratory distress syndrome (ARDS). On the other hand, the rise in cytokine levels associated with exposure to external agents is significantly hampered, probably because of persistently elevated pro-inflammatory cytokine secretion (4, 5). Patients with CS have a possibility for prolonged duration of viral infections and risk for superinfections leading to sepsis and increased mortality risk; this is especially relevant for hospitalized patients and mandates empirical prophylaxis with broad-spectrum antibiotics (6). Both COVID-19 and CS individually represent disease states of increased thromboembolic (TE) risk, requiring additional care (6).

Due to very limited data, it is still not possible to address these topics with certainty and make recommendations for optimal management of these patients. Current clinical practice guidance for management of CS during COVID-19 commissioned by the European Society of Endocrinology (ESE) emphasizes prompt and optimal control of hypercortisolism and adequate treatment of all comorbidities (7). Although individual circumstances must always be considered, we need more direct clinical experience, especially regarding the actual treatment of COVID-19 in this sensitive group. So far, there are only five published case studies of patients with CS and COVID-19, with eight patients in total (8–12). In this study, we present a patient with newly diagnosed ACTH-dependent CS who was diagnosed with COVID-19 before the initiation of specific medical treatment.

## Case Report

A 70-year-old female was admitted to our Covid hospital due to bilateral interstitial pneumonia caused by severe acute respiratory syndrome coronavirus 2 (SARS-CoV-2). Six days before she was discharged from endocrinology department of another hospital where she was hospitalized due to newly diagnosed diabetes mellitus. Her personal history was unremarkable, and she was vaccinated with two doses of inactivated COVID-19 vaccine Sinopharm BBIBP. During this hospitalization Cushingoid features were noted (moon face, centripetal obesity, thin extremities with multiple hematomas, bilateral peripheral edema), as well as diabetes mellitus (HbA1c 8.7%), arterial hypertension (BP 180/100 mmHg), hypokalemia (2.0 mmol/L), mild leukocytosis (WBC 12.9x10e9/L) with neutrophilia, and mildly elevated CRP (12.3 mg/L). Hormonal functional testing confirmed ACTH-dependent Cushing’s syndrome: morning ACTH 92.6 pg/mL (reference range 10-60 pg/mL), morning serum cortisol 1239 nmol/L (reference range 131-642 nmol/L), midnight serum cortisol 1241 nmol/L, lack of cortisol suppression in overnight dexamethasone suppression test (978 nmol/L). Pituitary MRI was unremarkable other than empty sella, and CT scan of thorax normal other than left adrenal hyperplasia. Diabetes mellitus was successfully controlled with metformin, hypertension with ACE-inhibitor, Ca-channel blocker and beta-blocker, and hypokalemia with potassium supplementation along with spironolactone. Steroidogenesis inhibitors were not available in this institution, but before referral to a tertiary care hospital she was tested for SARS-CoV-2, and the test came back positive (sample was obtained by nasopharyngeal swab). Since she was asymptomatic, with normal thoracic CT scan and stabile CRP level (9.1 mg/L), she was discharged with detailed recommendations for conduct in case of progression of COVID symptoms.

Next day she started feeling malaise with episodes of fever (up to 38.2°C). Symptomatic therapy was advised in an outpatient clinic (no antiviral therapy was recommended), but 5 days later respiratory symptoms ensued. During examination, the patient was weak, with dyspnea and tachypnea (RR 22/min), afebrile (36.9°C) and with oxygen saturation (SO_2_) of 85% measured by pulse oximeter. Chest X-ray confirmed bilateral interstitial pneumonia with parenchymal consolidation in the right lower lung lobe, so she was referred to the COVID hospital.

Laboratory analyses upon admission are presented in the **Supplementary Table 1**. In addition to her previous testing, elevated chromogranin A (CgA) level was verified (538.8 ng/mL, reference range 11-98.1). The patient was treated with supplemental oxygen with maximal flow of 13 l/min. For the reason of previously confirmed severe endogenous hypercortisolism, glucocorticoids were not administered. Due to limited therapeutic options and presumed further clinical deterioration, we decided to treat the patient with intravenous immunoglobulins (IVIg) 30 g iv for 5 days, starting from the 2^nd^ day of hospitalization. We did not observe any side effects of a given treatment. In parallel, the patient received broad-spectrum antibiotics (ceftazidime and levofloxacin), proton pump inhibitor, LMWH in prophylactic dose, oral and parenteral potassium supplementation along with spironolactone. She continued with her previous antihypertensive therapy with good control of blood pressure. While the patient was on oxygen supplementation, glycaemia was controlled with short acting insulin before meals. Following given treatment, we observed clinical, biochemical (**Supplementary Table 1**.) and radiological improvement (**Supplementary Figure 1**). Oxygen supplementation was gradually discontinued. With regard to D-dimer levels and risk factors for TE events due to COVID-19 and CS, we performed color Doppler scan of lower extremities veins, and CT pulmonary angiography, but there were no signs of thrombosis. During hospital stay, there were no signs of secondary infection and cotrimoxazole was not added to the current treatment. The patient was discharged with advice to continue her prior medical therapy along with increased dose of spironolactone and initiation of rivaroxaban. She was referred to the tertiary institution for the initiation of steroidogenesis inhibitor and further diagnostics.

## Discussion

Endogenous Cushing’s syndrome is a rare disease with an incidence of 0.7-2.4 million person-years in European population-based studies (13). Significant morbidity yields a standard mortality ratio of 3.7 (95%CI 2.3–5.3), with the highest mortality during the first year after initial presentation. COVID-19 pandemic imposes additional challenge to this fragile group of patients. Due to lack of solid experience, it is still difficult to define potential clinical course and outcome of patients with CS and COVID-19. In addition, currently there are no guidelines for management of SARS-CoV-2 infection in patients with active CS.

So far, only two small case series followed patients with Cushing’s disease (CD) in various disease stages (not all were active) during COVID-19 pandemic (9, 12). Small number of SARS-CoV-2 positive cases (3/22 and 2/61) is clearly biased by shortness of analyzed period (one and a half, and three and a half months). Additionally, a small number of patients was actually tested by nasopharyngeal swab for SARS-CoV-2 even in the presence of indicative symptoms, albeit mild. Nevertheless, all these limitations included, it seems that the prevalence of COVID-19 might be greater in patients with CD than in general population (12). This is accordant with studies on patients on exogenous glucocorticoid (GC) treatment. Overall, there is a growing body of evidence that patients on chronic GC therapy are at higher risk for SARS-CoV-2 infection and a severe course of disese, regardless of age and comorbidities (14). In many studies patients on high-dose GC therapy were at particularly high risk for a severe course of disease, so it is reasonable to assume that there is a dose-dependent effect (14).

All patients except one with endogenous CS and COVID-19 presented in literature were hospitalized, with majority of them requiring oxygen supplementation, which classified them as serious cases of disease (8–12). Parameters of inflammation (namely CRP) were highly variable (from normal to elevated) and did not seem to reflect severity of COVID-19 consistently. Two patients had fatal outcome; one with postoperative hypocortisolism that required stress doses of hydrocortisone, and with terminal kidney failure as significant comorbidity; the other with suspected EAS who developed ARDS in contrast to normal CRP and absence of fever (9, 12). Based on reported cortisol levels in these patients, it seems that the severity of COVID-19 pneumonia depended on severity of hypercortisolism (8–12). A patient with probable EAS even developed ARDS, which adds to ongoing controversy regarding the risk of ARDS due to SARS-CoV-2 in patients with CS (3, 15). We ourselves have treated a severely obese female patient with active CD on pasireotide, who developed ARDS despite addition of high doses of methylprednisolone (unpublished data). Additional risk imposed by comorbidities cannot be underestimated (15, 16). This is particularly relevant for obesity, that not only hampers immune system (leading to increased levels of IL-1, IL-6, and TNF-α), but adipocytes represent a reservoir of SARS-CoV-2 thanks to ACE2 receptor, crucial for virus attachment (15).

Majority of depicted patients with active CS were already medically treated for hypercortisolism but with various compliance (sometimes very poor), and two young patients have just started steroidogenesis inhibitors (metyrapone/ketoconazole). Infection with SARS-CoV-2 was treated by national protocols that were mostly based on supportive care. These protocols changed over time, so a few patients received antiviral therapy (favipiravir), and one young patient with suspected EAS was treated with methylprednisolone along with high doses of ketoconazole (10). Treatment was complicated with adrenal insufficiency (AI) in three patients (8, 11, 12).

We have presented a patient with CS and rapid development of serious case of COVID-19 pneumonia that required hospital admission and oxygen support. She was febrile and had positive laboratory parameters of inflammation. Her CS was active, with very high cortisol levels, no prior medical treatment and with clinical suspicion of EAS (ACTH-dependent disease of short duration, severe hypercortisolism, hypokalemia, very high CgA, no visible pituitary tumor). With this in mind, and with regard to rapid progression of COVID-19 pneumonia, it was our opinion that the patient required treatment with quick onset and presumable immune system modulation.

A logical approach to treatment of CS during COVID-19 pandemic includes meticulous therapy for comorbidities (namely antihypertensives, anti-diabetic drugs, low molecular weight heparin, etc.), and steroidogenesis inhibitors for treatment for hypercortisolemia (7). While some of these drugs demonstrate quick onset of action regarding normalization of cortisol level (and hence improve clinical comorbidities), rapid effects on immune system responses are not likely, which might be of great relevance in case of acute infection. Secondly, adrenolytic therapy increases a risk of AI, which can be even more perilous than CS in case of infection or other stress situations (8, 12, 15, 16). A modified “block and replace” approach may be considered, where addition of hydrocortisone could diminish the risk of AI (7). Still, there are a few potential pitfalls with this regimen as well. Some people fail to respond to high doses of adrenal-blocking agents due to genetic differences in the steroidogenic enzymes, since therapeutic responses to metyrapone and ketoconazole in patients with CS are associated with the polymorphism in the *CYP17A1* gene (17). Additionally, there are not enough data about possible interactions between adrenolytic drugs (majority of them being metabolized through the CYP450/CYP3A4 pathway) and medications used to treat COVID-19, most of which are only just emerging (18). Special concerns, amplified with similar potential effects of SARS-CoV-2 itself as well as specific therapies are liver dysfunction (metyrapone, ketoconazole), hypokalemia (metyrapone, ketoconazole), QT-interval prolongation (ketoconazole, osilodrostat), gastrointestinal distress (mitotane, osilodrostat, etomidate) (18). Metyrapone may cause accumulation of androgenic precursors secondary to the blockade of cortisol synthesis, that can virtually enhance expression of transmembrane protease serine 2 (TMPRSS2), found to be essential to activate the viral spikes, induce viral spread, and pathogenesis in the infected hosts (19). Another important issue concerns biochemical estimation of disease control (and hence risk for AI), since most commercially available assays can overestimate cortisol level in patients treated with metyrapone due to cross-reactivity with the precursor 11-deoxicortisol (7, 15). Mass spectrometry is a method of choice to overcome this problem, but it is not available in many centers. Some centers advocate titration and/or temporary halting medical therapies in the treatment of patients with CS in the context of COVID-19 infection (20). Treatement was stopped in a few patients with severe COVID-19 symptoms who were then given high dose GC for a few days with no long-term complications, and with full recovery (20).

There are no data about the effect of anti-viral drugs in patients with CS and COVID-19. A special concern refers to adipose tissuse, as adipose tissue is difficult for antiviral drugs to reach. It cannot be excluded that the constant release of viral replicas from the adipose tissue reservoir may interfere with COVID-19 infection treatment, delaying its resolution and favoring a worse prognosis (15). If antiviral drugs are started, it is suggested that immunocompromised patients may require prolonged therapy (18). However, the timing is difficult in practice and candidates for antivirals are limited.

Since the clinical course of COVID-19 only initially depends on viral replication, immunomodulatory therapy emerged as a valuable treatment option to control the host immune response. This became apparent ever since RECOVERY trial proved efficacy of glucocortiods (21). But this therapeutic option is fairly inapplicable in patients with active CS, since glucocorticoid treatment in chronic hypercortisolism seems to enhance immune system alterations (22). In parallel with the development of new agents, it is prudent to study the efficacy of existing therapeutic options with acceptable safety profile (20). Beside glucocorticoids, inflammation blockers, intravenous immunoglobulin and convalescent plasma were used in various settings (23).

Intravenous immunoglobulin (IVIg) is a blood product prepared from the serum pooled from thousands of healthy donors, containing a mixture of polyclonal IgG antibodies, mostly IgG1 and IgG2 subclasses (24, 25). Initial rationale for its use was immunodefficiency due to hypoglobulinemia. Since then it has been shown that IVIg exerts pleiotropic immunomodulating action involving both innate and adaptive immunity and it has been used in a variety of diseases (26). In previous studies on MERS (Middle East Respiratory Syndrome) and SARS (Severe Acute Respiratory Syndrome) using IVIg showed beneficial clinical effects (25). Although pathogenesis of COVID-19 has not be fully elucidated, there is a consensus that immune-mediated inflammation plays an important role in the progression of this disease, just as it did in prior coronavirus infections (27). In this context, the actual role of IVIg in COVID-19 patients might be not to boost the immune system, but through its immunomodulatory effect to suppress a hyperactive immune response that is seen in some patients (28). So far, a limited number of studies, case series and meta-analyses demonstrate a promising potential of IVIg in patients with COVID-19. The effect was demonstrated in terms of mortality, improvement of clinical symptoms, laboratory examinations, imaging and length of hospital stay, especially in patients with moderate/severe form of the disease, and with emphasis on early administration (within 3 days of admission) (24, 25, 27–31). A recent double blind, placebo-controlled, phase 3, randomized trial tested hyperimmune intravenous immunoglobulin (hIVIg) to SARS-CoV-2 derived from recovered donors with no demonstrated effect compared with standard of care, but therapy was administered in patients symptomatic up to 12 days (32). Additional clinical trials are underway, hopefully with more guidance for proper selection of patients that might benefit from this type of treatment.

## Conclusion

To our knowledge, this is the first case of IVIg treatment in a COVID-19 patient with CS. It is our opinion that immune-modulating properties of IVIg might present an attractive treatment option, especially in those CS patients that show rapid clinical progression and positive laboratory parameters of inflammation. While we await for new therapeutic modalities for COVID-19 and while some of the modalities remain not widely available, IVIg is more accessible, safe method, which could be rescuing in carefully selected patients. Of note, we consider our patient’s vaccinal status as an unquestionable positive contributor to the favorable outcome

## Data Availability Statement

The raw data supporting the conclusions of this article will be made available by the authors, without undue reservation.

## Ethics Statement

Ethical review and approval was not required for the study on human participants in accordance with the local legislation and institutional requirements. The patients/participants provided their written informed consent to participate in this study. Written informed consent was obtained from the individual(s) for the publication of any potentially identifiable images or data included in this article.

## Author Contributions

BP, AS, JV, TG, MJ-L, JV, VS, ZG and TA-V analyzed and interpreted the patient data. BP, AP, DI, and DJ were major contributors in writing the manuscript. All authors contributed to the article and approved the submitted version.

## Conflict of Interest

The authors declare that the research was conducted in the absence of any commercial or financial relationships that could be construed as a potential conflict of interest.

## Publisher’s Note

All claims expressed in this article are solely those of the authors and do not necessarily represent those of their affiliated organizations, or those of the publisher, the editors and the reviewers. Any product that may be evaluated in this article, or claim that may be made by its manufacturer, is not guaranteed or endorsed by the publisher.
