# Supplementary material for: Successful Immunomodulatory Treatment of COVID-19 in a Patient With Severe ACTH-Dependent Cushing’s Syndrome: A Case Report and Review of Literature
Source: Front Endocrinol (Lausanne). 2022 Jun 22;13:889928. doi: 10.3389/fendo.2022.889928 (PMC9257249; doi:10.3389/fendo.2022.889928)
Supplement: Supplementary file 1 [file DataSheet_1.pdf]

Supplementary Material

1.1 Table 1: Blood test results upon admission and during hospitalization

	06/10/2021	09/10/2021	12/10/2021	16/10/2021
**CRP (0.0-8.0 mg/L)**	183.1	60.6	18.2	7.6
**White blood cells (4.40-11.00x10e9/L)**	9.0	4.8	5.5	7.5
**Neutrophil count (2.1-6.50x10e9/L)**	7.73	3.59	4.10	5.75
**Lymphocyte count (1.20-3.40x10e9/L)**	1.08	0.93	0.96	1.25
**Hemoglobin (119-157 g/L)**	135	103	125	121
**Platelets (158-424x10e9/L)**	144	138	224	255
**D-dimer (cut off 0.5 mg/L FEU)**	1.85	1.45	1.67	1.81
**Fibrinogen (2.0-4.0g/L)**	7.0	4.6	4.3	4.1
**Glucose (3.9-6.1 mmol/L)**	7.6	5.2	7.8	7.4
**Urea (2.5-7.5 mmol/L)**	8.3	5.6	5.7	6.9
**Creatinine (45-84 umol/L)**	64	57	47	55
**Proteins (62-82 g/L)**	61	53	64	56
**Albumin (34-55 g/L)**	33	23	25	24
**Sodium (135-148 mmol/L)**	143	147	145	147
**Potassium (3.5-5.1 mmol/L)**	2.4	2.2	3.4	3.7
**GPT (0-37 U/L)**	47	31	64	54
**GOT (0-41 U/L)**	66	36	79	80
**ALP(40-120 U/L)**	148			149
**GGT (0.38 U/L)**	247			346
**LDH (220-460 U/L)**	1485	911		898
**IL-6 (0.00-3.40 pg/mL)**	31.0			
**Ferritin (4.6-204 ug/L)**	3160.6			1869.2

CRP, C-reactive protein; GPT, glutamic pyruvic transaminase; GOT, glutamic oxaloacetic transaminase; ALP, alkaline phosphatase; GGT, gamma-glutamyl transferase; LDH, lactate dehydrogenase; IL-6, interleukin-6

1.2 Supplementary Figures

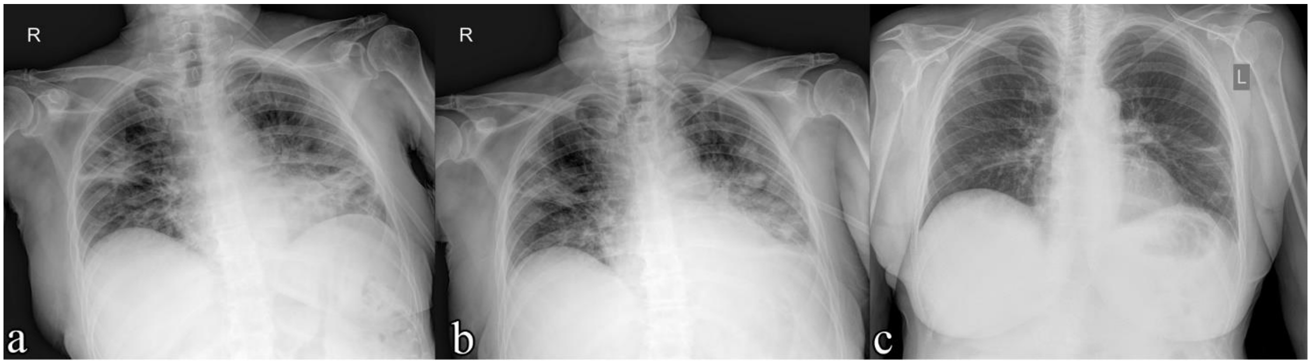

Figure 1. a) Chest X-ray on admission, b) after 5 days of treatment, c) 2 weeks after discharge
